# The Relationship between Carotid Doppler Ultrasound and EEG Metrics in Healthy Preschoolers and Adults

**DOI:** 10.3390/brainsci10100755

**Published:** 2020-10-20

**Authors:** Galina V. Portnova, Aleksandra V. Maslennikova, Elena V. Proskurnina

**Affiliations:** 1 Laboratory of the Human Higher Nervous Activity, Institute of Higher Nervous Activity and Neurophysiology of the Russian Academy of Sciences, 117485 Moscow, Russia; alexm2004@list.ru; 2 Laboratory of Molecular Biology, Research Centre for Medical Genetics, 115522 Moscow, Russia; proskurnina@gmail.com

**Keywords:** carotid doppler ultrasound, EEG, power spectral density, fractal dimension, peak alpha frequency, healthy preschoolers, healthy adults, children

## Abstract

Despite widespread using electroencephalography (EEG) and Doppler ultrasound in pediatric neurology clinical practice, there are still no well-known correlations between these methods that could contribute to a better understanding of brain processes and development of neurological pathology. This study aims to reveal relationship between EEG and Doppler ultrasound methods. We compared two cohorts of adults and preschool children with no history of neurological or mental diseases. The data analysis included investigation of EEG and carotid blood flow indexes, which are significant in neurological diagnosis, as well as calculation of linear and non-linear EEG parameters and ratios between the systolic peak velocities of carotid arteries and carotid blood asymmetry. We have found age-dependent correlations between EEG and power Doppler ultrasound imaging (PDUI) data. Carotid blood flow asymmetry correlated with delta-rhythm power spectral density only in preschoolers. The ratios of blood flow velocities in the internal carotid arteries to those in the common carotid arteries correlated with higher peak alpha frequency and lower fractal dimension; moreover, they were associated with lower Epworth sleepiness scale scores. The study revealed significant correlations between EEG and PDUI imaging indexes, which are different for healthy children and adults. Despite the fact that the correlations were associated with non-clinical states such as overwork or stress, we assumed that the investigated parameters could be applicable for clinical trials.

## 1. Introduction

Transcranial Doppler ultrasonography and electroencephalography are widely used in clinical practice. In pediatric neurology, these methods are the basis for diagnosing neurological and developmental diseases [1,2]. However, there are still no well-known correlations between electroencephalography (EEG) and Doppler ultrasound data that could contribute to a better understanding of brain processes and development of neurological pathology.

In pediatric neurology, the variability of power Doppler ultrasound imaging (PDIU) data was earlier referred to the developmental problems in preschoolers [3]. The most significant impact of ultrasound findings, especially for speech development, was related to blood flow asymmetry [4]. The other correlation between children’s neurological status and ultrasound indexes has been associated with velocity ratios in carotid and cerebral arteries. Abnormalities in the cerebral blood flow have been considered as crucial contributors to the pathogenesis of periventricular white matter lesions in preterm infants [5]. Furthermore, cerebral blood flow (CBF) has been correlated with cognitive function deficit in attention deficit hyperactivity disorder (ADHD) and autism spectrum disorders (ASD) [6]. In particular, the cortical hemodynamic responses in children with ADHD has been associated with working memory deficits [7]. Significantly higher regional cerebral blood flow (rCBF) values have been detected throughout the frontal white matter and subcortical gray matter in subjects with ASD [8,9]. These parameters were lowered with increasing IQ in a typically developing control group; such a correlation was absent in participants with ASD whose values were elevated across all IQ levels [10].

Little information is available on the EEG abnormalities that could be associated with developmental problems and correlated with CBF. Some studies have demonstrated contradictory results and have failed to replicate the most encouraging differences between theta or theta-beta band power ratios between children with ADHD and typically developing children [11] and adults [12]. In addition, the predominance of slow-wave activity in children with developmental disorders as well as neurological or mental impairments remained most promising [13]. Recent studies have demonstrated that the increase in the delta band to other band ratios was associated with ADHD syndrome [14] and could be predicted by EEG connectivity in delta and gamma bands [15,16].

There is reason to assume a relationship between EEG parameters and carotid Doppler ultrasound indices in adults. Thus, significantly lower peak alpha frequency (PAF) was found in individuals with traumatic brain injury after task solving [17]. Furthermore, reduction in PAF around the motor cortex region was associated with physical fatigue [18]. Similar correlations were also revealed for fractal dimension (FD) increases in healthy adults. For example, higher FD was associated with higher emotional reactivity [19,20] or emotional arousal [21,22] during emotional stress, and such a correlation was also found in sleep-deprived subjects during car driving [23]. Other data have indicated that increased FD was associated with fatigue in adults; such a correlation was strengthened by aging [24,25]. Thus, the decrease in the internal carotid artery/common carotid artery (ICA/CCA) velocity ratio in healthy adults was associated with mental and physical exhaustion because of stress.

In our study, we aimed to find relationship between PDUI and EEG data, assuming that they varied between adults and children. We selected the indicators of blood flow asymmetry and ratios between systolic peak velocities of carotid arteries obtained using PDUI and EEG power spectral density or fractal dimension of EEG, which was more sensitive to changes in EEG in cases of mental and neurological impairments in adults [26].

## 2. Materials and Methods

### 2.1. Participants

Twenty-nine children 4–6 years old. (5.1 ± 0.8, 17 boys, 12 girls) and 45 adults 20–40 years old (31.7 ± 1.9, 20 men, 25 women) participated in our study. The larger group size of adults was due to age heterogeneity. None of the participants had previously any psychiatric, neurological disorders or head trauma. They also were not taking any psychiatric medication at the time of the study. This study was approved by the Ethics Committee of Institute of Higher Nervous Activity and Neurophysiology of the Russian Academy of Sciences (RAS). All healthy voluntaries (or their parents) provided their written informed consent after receiving a complete description of this study.

Adults were asked to complete the Epworth Sleepiness Scale (ESS) and The Stanford Sleepiness Scale (SSS) to exclude sleepiness. The inclusion criteria were as follows: ESS scores 0–12 (6.7 ± 1.4 averaged score), SSS scores 1–4 (1.6 ± 0.7 averaged score), no history of neurological or mental disease.

Children were tested by clinical psychologist to exclude the cognitive or mental impairment; parents were asked to complete the CARS (Childhood Autism Rating Scale) to exclude ASD. Children with a CARS score of 25 or higher were not included on the study.

Participants were informed about the experimental procedure, and the study was conducted in accordance with the Helsinki Declaration, while the study protocol was approved by the Ethics Departments of the Institute of Higher Nervous Activity and Neurophysiology of RAS. All healthy participants and patients’ legal representatives provided written informed consent.

**Figure 1:**
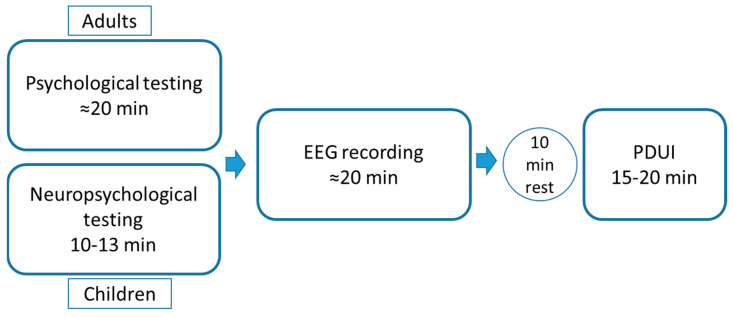
Schematic design of the study.

A schematic design of the study is presented in Figure 1.

### 2.2. EEG and Doppler Examination

#### 2.2.1. Procedure

The study was performed from 2015 to 2019 at the Rehabilitation Center for Children, Moscow, Russia in collaboration with the Institute of Higher Nervous Activity and Neurophysiology RAS. The examinations were performed at the same time—at 10:00–12:00. Before EEG recording and PDUI, the psychological testing (10–15 min) was performed. We recorded resting state EEG with eyes closed and eyes opened (5–7 min). As children could not remain motionless for this time, we asked them to close eyes only for a 10–30 s. However, for EEG analysis in adults, we considered the same intervals as for the children—the first 30 s. After a 10-min break following the EEG examination, the transcranial Doppler ultrasonography was performed.

#### 2.2.2. EEG Recordings

During EEG recording, the participants sat in a comfortable position after being instructed to remain calm with their eyes closed. We recorded resting state EEG (rsEEG) (sampling rate of 250 Hz) for 15 min using the EEG amplifier “Encephalan” (Medicom MTD, Taganrog, Russian Federation) with 19 AgCl electrodes placed according to the International 10–20 System. The electrodes placed on the left and right mastoids served as joint references under unipolar montage. The vertical electrooculogram (EOG) was measured with AgCl cup electrodes placed 1 cm above and below the left eye canthus, the horizontal EOG was measured with electrodes placed 1 cm lateral from the outer canthi of both eyes. The electrode impedances were less than 10 kΩ.

#### 2.2.3. Doppler Ultrasonography of Head and Neck Vessels

The GE Healthcare LOGIQ 9 Ultrasound scanner was used for color Doppler ultrasonography with linear probes for each patient in the supine position after a 10-min rest. The probe operates at two different frequencies: 9 and 13 MHz. Images of the common carotid were captured at 9 MHz, and the internal and external carotid images were captured at 13 MHz. Pulse wave Doppler measures are not affected by the probe frequency setting. Ultrasound scans are recorded to super VHS videotape for analysis. We evaluated structural conditions of the vessels, systolic/diastolic linear blood flow rates (BFRs) and pulsatility indexes. According to our hypothesis, we calculated the following: (1) the ratio between the systolic peak velocities of the internal and common carotid arteries (ICA/CCA); (2) the ratio between the systolic peak velocities of the internal and external carotid arteries (ICA/ECA); (3) the asymmetry of the blood flow in carotid arteries (AC), which was calculated as $\surd \frac{{\left(rICA-lICA\right)}^{2}}{{\left(rICA+lICA\right)}^{2}}$ (for internal carotid artery).

### 2.3. EEG Data Analysis

#### 2.3.1. EEG Preprocessing

Continuous EEG fragments of each subject were cleaned from artifacts caused by eye movements and blinks by the Medicom plugin using electrooculogram (EOG) data. EOG and muscle artifacts were identified with independent component analysis provided with MATLAB. Movement artifacts were detected and excluded manually. The continuous EEG of each subject was filtered with band-pass filter 0.5–30 Hz. After artifact reduction, the 5 min EEG fragments (317.9 ± 28.6 ms) with 19 channels according to the international 10–20 system were used for further analysis.

#### 2.3.2. Power Spectral Density (PSD)

Additionally, the fast Fourier transform was used to analyze PSD. The resulting normalized spectra were integrated over intervals of unit width in the range of interest (delta band: 2–4 Hz, theta1 band: 4–6 Hz, theta2 band 6–8 Hz, alpha1 band 8–10 Hz, alpha2 band 10–12 Hz, alpha 3 band 12–14 Hz and beta band 14–20 Hz) in accordance with filtering of non-linear features.

#### 2.3.3. Fractal Dimension (FD)

We performed the calculations of the examined signal bandpass-filtered in the range of interest (1.6–30 Hz), Butterworth 12th order filter was used. FD was evaluated using the Higuchi algorithm [24].

#### 2.3.4. Peak Alpha Frequency (PAF)

An average PAF value for each recording was defined as the discrete frequency that has the highest magnitude within the range of 7–13 Hz in each recording for each of the 19 channels.

#### 2.3.5. EEG Asymmetry Index

To compute EEG asymmetry on our data, we used the modified EEGLAB plugin calculated EEG asymmetry index for the range of interest using the following formula: mean(log(PSD_R)–log(PSD_L)).

### 2.4. Statistical Analysis

A repeated-measures ANOVA with Bonferroni correction for multiple comparisons (*p* < 0.05) and Mann–Whitney test were used to determine group effects on EEG metrics (post hoc Tukey). We analyzed a possible relationship of the EEG metrics with the and ultrasound parameters and rates of psychological assessment using Spearman correlation analysis corrected for multiple comparisons by the clustering method with 500 permutations at each node (the Bonferroni corrected *p*-value of 0.05; Matlab toolbox for Вrain Computer Interface). The permutation test was performed to compensate for the multiple statistical estimations of the correlations in different EEG channels. The correlation for each EEG channel was computed with Spearman correlation across subjects. Only significant (*p* < 0.05) correlation values were used for further analysis.

## 3. Results

### 3.1. EEG Data

PSD. The 6–8 Hz PSD in the group of children was significantly higher compared to adults, and 10–12 Hz PSD was higher in the group of adults compared to children (*F*(1, 72) = 13.5583, *p* = 0.00021). The difference was most pronounced in the center-parietal areas.

FD. The significant differences were not detected.

PAF. The PAF was significantly higher in the group of adults compared to children in parietal and occipital areas (*F*(1, 72) = 9.1863, *p* = 0.00389).

EEG asymmetry index. The EEG asymmetry index was significantly higher in the group of children compared to adults in parietal and occipital areas (*F*(1, 72) = 9.1863, *p* = 0.00389) for alpha1-band PSD. Other significant differences were not detected.

### 3.2. Ultrasound Data

The systolic/diastolic linear BFRs and pulsatility indices corresponded to normative values in both groups of subjects. The subjects with structural abnormalities of carotid vessels were excluded from the investigation (Table 1). The mean values of parameters of interest (ratios and asymmetry index) are depicted in Table 1. All measures were significantly higher in children compared to adults (*z* > 2.6, *p* < 0.01).

### 3.3. Correlations between EEG and Ultrasound Data

#### 3.3.1. Correlations between EEG and Ultrasound Data in Children

**Figure 2:**
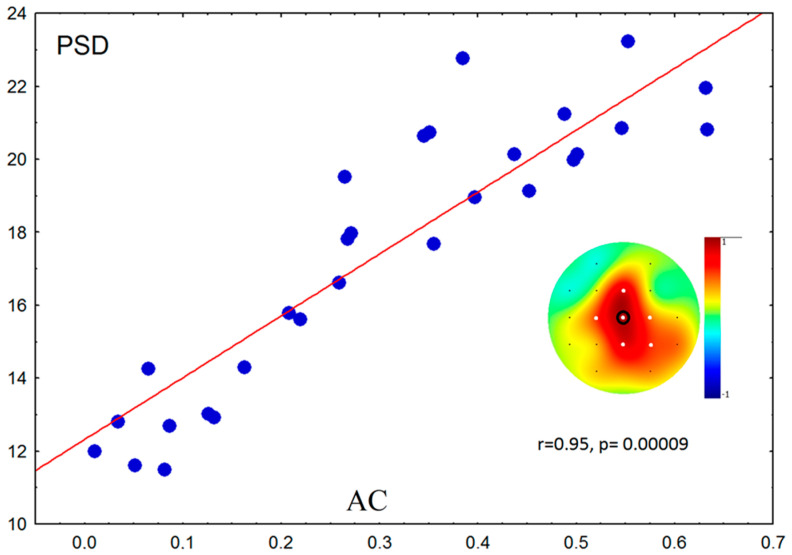
Scatterplot of power spectral density (PSD) data (2–4 Hz) against asymmetry of the blood flow in carotid arteries (AC) in children. White dots—significant correlations after Bonferroni correction and permutation test; black circle—electrode used for scatterplot.

More prominent asymmetry of ICA (AC) in children correlated significantly with higher slow-wave activity in the central area (supported by the permutation test) (Figure 2).

#### 3.3.2. Correlations between EEG and Ultrasound Data in Adults

**Figure 3:**
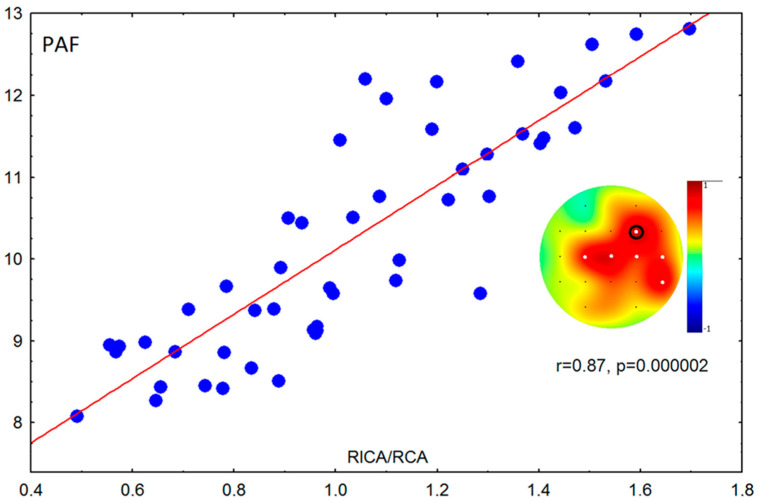
Scatterplot of peak alpha frequency (PAF) against ratio between the systolic peak velocities of the right internal and common carotid arteries (RICA/RCCA) in adults. White dots—significant correlations after Bonferroni correction and the permutation test; black circle—electrode used for scatterplot.

The ratio of the right ICA/CCA correlated with PAF of the right hemisphere in group of adults (supported by the permutation test); a similar effect was found for the left ICA/CCA but did not remain significant after Bonferroni correction. The ratio of the left ICA/CCA negatively correlated with FD of the left hemisphere in the group of adults (supported by permutation test); a similar effect was found for the right ICA/CCA but did not remain significant after Bonferroni correction (Figure 3).

#### 3.3.3. Correlations with Age, Gender, and Questionnaires

**Figure 4:**
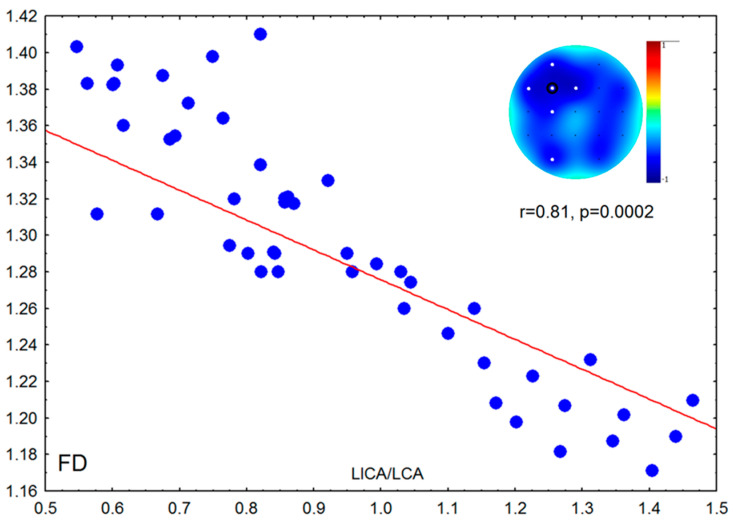
Scatterplot of fractal dimension (FD) against the ratio between the systolic peak velocities of the left internal and common carotid arteries (LICA/LCCA) in adults. White dots—significant correlations after Bonferroni correction and the permutation test; black circle—electrode used for scatterplot.

The ICA/CCA ratio in adults negatively correlated with ESS scores (*r* = −0.43, *p* = 0.0007). The PAF in the group of children correlated with age (*r* = 0.41, *p* = 0.001) (see Figure 4). Other correlations did not pass Bonferroni correction.

## 4. Discussion

Power Doppler ultrasound imaging of carotid arteries of patients in acute states could provide evidence of brain damage that is also registered by EEG. In particular, in the case of brain death, atypical blood flow velocity patterns of common carotid arteries characterized by involvement of a single systolic peak and a marked reverse flow component have been revealed [27,28]. Ipsilateral EEG suppression has been related to a regional cerebral blood flow (CBF) mean velocity threshold lower than 15 cm/s [29]. Other studies have also reported on the significant dynamics of the velocity of carotid arteries associated with brain ischemia and correlated with variable EEG changes [30]. In particular, the most valuable impact was associated with the velocity of the internal carotid artery, in which occlusion induced slowing of the EEG in patients with cerebral ischemia [31].

Despite high individual variability in the diameter and velocities of carotid arteries [32,33], some normative values for carotid flow rates [34] could indicate aging, development, or vascular disease [35,36]. For example, the ratio of peak velocities between internal and common carotid arteries evolves during maturation [36,37]. Its sensitivity to pathologies attributed to the EEG dynamic could also change during maturation and aging. The origin of age-depended changes could be in considerable differences of brain activity of adults and children. First, mental and cognitive functions are already formed in adults, and carotid blood flow influences them differently to children [38,39,40]. Second, the adult brain is much less capable of developing any plastic changes and is more prone to the development of ischemic disorders as a result of atherosclerosis and hypertension development [41].

Numerous studies have investigated the effect of the ICA/CCA velocity ratio on the risks of developing coronary disease or other vascular pathologies that can cause cognitive impairments. Some findings [42,43] point to specific metabolism connected with blood flow in schizophrenics, such as CBF for cognitive task word-face recognition. Confirming these assumptions, the flow rate ratios between internal and common carotid arteries have been previously associated with the development of atherosclerosis and thrombosis at human carotid artery bifurcations; it was found that the size of the recirculation zone during ischemia was largely dependent on this ratio [44]. It could serve as a useful parameter in screening patients with suspected internal carotid artery stenosis [45].

Here, we have studied the following EEG features: (1) power spectral density in delta band: 2–4 Hz, theta1 band: 4–6 Hz, theta2 band 6–8 Hz, alpha1 band 8–10 Hz, alpha2 band 10–12 Hz, alpha 3 band 12–14 Hz and beta band 14–20 Hz; (2) fractal dimension in the range of interest (1.6–30 Hz); (3) peak alpha frequency; and (4) EEG asymmetry index. Gamma band (from 30 to 80–100 Hz) was not investigated because of strong interference by muscle artifacts and minor eye movement. However, the study of gamma rhythm is of great interest since it is influenced by various types of cognitive processes. In the future, we are going to study gamma activity using magnetoencephalography. In accordance with the assumption that carotid blood flow indices could be normally changed during maturation and aging, our findings demonstrated that ICA/CCA ratios were sensitive to the EEG features only in adults, whereas CBF asymmetry correlated with higher slow-wave activity only in children. However, the origin of these age-specific associations between blood flow velocity indices and EEG metrics could be consisted not only in age-related changes. In particular, decrease in velocity ratio between internal and common carotid arteries was associated with PAF decrease and FD increase. These EEG features were associated with cognitive and mental processes in healthy subjects and groups of patients [46]. Thus, lower PAF has been previously associated with specific cognitive and mental states related states correlated with the overfatigue of increased activity. In fact, questionnaires and self-reports demonstrated that subjects with the lowest ICA/CCA velocity ratios reported symptoms of chronic fatigue, excessive exercise, and episodes of sleep disturbance.

Asymmetry of the carotid blood flow (AC) in our study was detected predominantly in children and correlated with slow-wave activity (2–4 Hz). Previous findings concerning normative intracranial arterial velocities also demonstrated blood flow asymmetry in children [47]. Physiological hemispheric asymmetry of cerebral blood resulting from the functional asymmetry of the brain in normal preschool children has been observed. Such asymmetry is especially marked in newborns. It manifests itself in an increase in blood flow and metabolism in the right hemisphere because of immaturity in the left hemisphere of newborns [48]. As children grow, the blood flow asymmetry in the basins of the right and left internal carotid arteries remains until school age [49].

In addition, asymmetry of the blood flow could induce asymmetric perfusion variable tissues [50] and have a high impact on brain activity. It was also associated with asymmetry of cerebral ischemia in children with Sturge–Weber syndrome [51] and in children with ADHD [52,53]. Moreover, asymmetry of the blood flow has been associated with language and handedness. Anomalous hemispheric specialization was detected in children with autism [54] and patients with schizophrenia [55].

Thus, blood flow asymmetry in children could be interpreted as either normal maturation or pathology. The correlation with the higher delta-band also supports both assumptions. In particular, according to previous studies, delta waves could normally be detected in preschool children and became more pronounced the younger the children [56]. Furthermore, higher delta rhythm power could also indicate abnormal development in children [57]. Intermittent rhythmic delta activity was shown to be higher in children with epilepsy [58,59,60] and Down syndrome [61]. In addition, other studies have attributed slow-wave activity to central nervous system lesions [62] and metabolic derangement appearing after stroke [63,64], which correlated with infarct volume [65] and brain function in this group of patients [66].

In summary, our findings demonstrated that the correlation between the delta-rhythm PSD and age did not pass the Bonferroni correction (*p* = 0.04). They also showed that the correlation between AC asymmetry did not attain statistical significance (*p* = 0.07). In addition, we cannot exclude the possibility that the correlation between blood flow asymmetry and the delta band was associated with both the normal process of brain maturation and pathological dysfunctions, such as ADHD. We assumed that the revealed correlations between EEG and PDUI parameters obtained from healthy children and adults were probably associated with aging, development-related processes, and changes associated with fatigue or mental stress. Nevertheless, we hypothesized that the identified EEG and AC indices would have a significant impact for different groups of patients and should be considered.

## 5. Conclusions

The present exploratory study presents a possible correlation between cerebral hemodynamics and EEG parameters for children depending on their age. The correlation in the velocity ratio between internal and common carotid arteries was associated with the parameters of the EEG alpha-rhythm frequency and fractal dimension, which have been previously associated with stressfulness and overfatigue in healthy adults.

## Figures and Tables

**Table 1:** Descriptive statistics of the ultrasound data.

	Valid N	AC *	Left		Right	
			ICA/ECA **	ICA/CCA ***	ICA/ECA	ICA/CCA
Children	29	0.21 ± 0.1	1.05 ± 0.2	1.24 ± 0.2	1.00 ± 0.14	1.14 ± 0.3
Adults	45	0.11 ± 0.1	0.90 ± 0.2	0.89 ± 0.2	0.81 ± 0.15	0.93 ± 0.2

* AC—asymmetry of the blood flow in carotid arteries. ** ICA/ECA—the ratio between the systolic peak velocities of the internal and external carotid arteries. *** ICA/CCA—the ratio between the systolic peak velocities of the internal and common carotid arteries.
